# Perceptions and attitudes of medical oncologists regarding fertility preservation and pregnancy in high‐risk cancer patients: A survey among Canadian medical oncologists

**DOI:** 10.1002/cam4.5023

**Published:** 2022-07-14

**Authors:** Bader Alshamsan, Igal Kushnir, Sulaiman Al‐Saadi, Amirrtha Srikanthan

**Affiliations:** ^1^ Division of Medical Oncology The Ottawa Hospital Cancer Centre Ottawa Ontario Canada; ^2^ Sackler Faculty of Medicine Tel Aviv University Tel Aviv Israel; ^3^ Department of Medicine The University of Ottawa Ottawa Ontario Canada; ^4^ Ottawa Hospital Research Institute Ottawa Ontario Canada

**Keywords:** chemotherapy, fertility preservation, medical oncologists, pregnancy

## Abstract

**Purpose:**

Infertility is a critical late toxicity that impacts adolescent and young adult (AYA, ages 15–39 years) cancer survivors. International oncology societies recommend discussing fertility preservation (FP) for all AYA patients, regardless of stage or prognosis. We aim to understand Canadian medical oncologists' perceptions, attitudes, and knowledge toward FP and pregnancy in patients with cancer, including advanced stages and high risk for recurrence.

**Methods:**

An anonymous electronic survey utilizing hypothetical scenarios was sent to medical oncologists in the province of Ontario, Canada. Descriptive statistics were used to summarize all data. Logistic regression models were constructed to identify factors that predicted FP discussions and referrals.

**Results:**

The survey was received by 91 medical oncologists, and the response rate was 44%. Fifty‐eight percent of respondents offer FP for all patients. Physicians are more likely to refer patients for FP before curative intent therapy than before palliative chemotherapy (95% vs. 39.5%, *p* < 0.001). Most respondents (86%) are comfortable discussing FP; however, only 31% self‐reported feeling up‐to‐date on knowledge of current FP methods. Female physicians were more likely to report up‐to‐date knowledge and confidence discussing FP with patients. Forty percent of respondents identified that concerns about the welfare of the resulting offspring should not be a cause for denying patients assistance in reproduction.

**Conclusion:**

There is a significant difference in physician attitude toward offering FP based on the cancer stage. Increased awareness of standard of care guidelines and resources for difficult situations may improve the frequency of discussions about FP in motivated cancer patients.

## Graphical abstract

Adolescents and young adults with cancer are inconsistently offered fertility preservation (FP) discussions prior to treatment. Differences in physician attitude toward offering FP discussions exist based on the cancer stage.

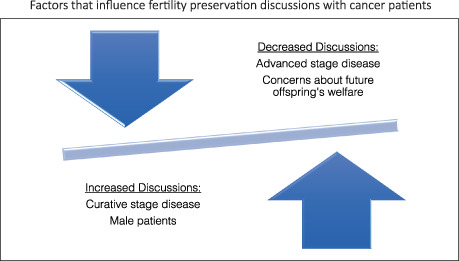

## 1 BACKGROUND

An essential concern for the majority of adolescents and young adults (AYA, ages 15–39 years)^1^ affected by cancer is future fertility.^2^ The risk of infertility from cancer therapy can be up to 50%–80% in males and females, respectively, depending on tumor type, age at treatment, and types of treatments used.^3^, ^4^ Many AYA will require anti‐cancer therapies that can have a detrimental impact on fertility.^5^ Other therapies that do not impact fertility may need to be taken for prolonged periods, during which procreation is contraindicated.^6^, ^7^, ^8^ During this time, fertility reductions may occur due to the natural effect of aging, particularly in female patients.^9^, ^10^ With the increasing incidence of cancers in individuals of reproductive age and their decreasing mortality rates,^11^, ^12^ ensuring fertility is addressed is of increasing importance.

Recognizing the importance of this problem, the American Society of Clinical Oncology (ASCO) first published guidelines in 2006 recommending infertility risk discussion with cancer patients.^13^ In 2013, these guidelines were updated to recommend that discussions explaining the risk of infertility as a consequence of cancer treatment be undertaken with all newly diagnosed cancer patients of reproductive age.^14^ Furthermore, in 2018, the guidelines were updated to provide current guidance regarding fertility preservation (FP) referrals to motivated patients.^15^

Despite national,^16^ and international^17^ recommendations supporting the fertility and FP discussions prior to treatment, many patients do not receive adequate information on FP.^18^, ^19^, ^20^, ^21^ More than half of AYA cancer patients post‐treatment report that they did not receive counseling regarding the impact of cancer therapy on fertility.^19^, ^22^, ^23^ While the subject of FP for cancer patients has been discussed in several publications,^2^, ^3^, ^13^, ^18^, ^24^ patients with cancer at elevated risk for recurrence and metastatic cancer were not explicitly addressed. Patients of reproductive age with metastatic cancer pose several unique clinical challenges and ethical dilemmas. These challenges include the risk of death to the patient and potential impacts on the ability to carry a pregnancy to term safely. Although the Ethics Committee of the American Society for Reproductive Medicine endorses that a patient's prognosis or concerns about the welfare of resulting offspring should not be a cause for denying cancer patients assistance in reproduction,^25^ some oncologists disagree.^18^

In this study, we establish the current perceptions and attitudes among Canadian medical oncologists toward FP and pregnancy risk for a patient with a focus on patients undergoing treatments for high‐risk localized cancer and patients with metastatic cancer.

## 2 METHODS

An anonymous electronic survey was administered to Canadian physicians. Eligible study participants included Canadian medical oncologists, medical oncology fellows, and residents who treat cancer patients in the province of Ontario, Canada's most populous province.^26^

### 2.1 Survey development

The survey was developed by two clinicians and one researcher with experience in oncology management, AYA with cancer, and survey development. Prior to survey distribution, face and content validity were assessed by pilot testing the survey among ten physicians who treat oncology patients in a focus group at The Ottawa Hospital. Refinements were made to the survey based on consensus in this focus group and approved by the Research Ethics Board prior to distribution. Survey completion took approximately 20 min.

### 2.2 Survey structure

The survey included one question for eligibility and 44 questions divided into three major sections (Appendix S1). The first section collected participants' demographic data, including sex, marital status, parity, religion, years of practice in medical oncology, tumors sites treated, and type of workplace (academic or community center). The second section established the participants' prior experience with FP, including the number of patients referred to FP annually, the number of new consults in the last year for AYA patients, attitudes toward FP prior to chemotherapy with all childbearing ages, concern about future offspring welfare, comfort with FP counseling and self‐reported knowledge base with respect to current FP methods. The third section included four scenarios: (a) curative intent chemotherapy for a female with locally advanced triple‐negative breast cancer (BC) and pregnancy after 6 months of chemotherapy, (b) curative intent chemotherapy for a male with testicular cancer, (c) palliative intent chemotherapy for a male with gastric cancer, and (d) palliative intent second‐line chemotherapy for a female with metastatic Ewing sarcoma. With each scenario, questions were posed regarding attitudes toward FP prior to chemotherapy, awareness of provincial coverage for FP, and personal opinions for each scenario. Theoretical scenarios were provided which aimed to assess physician attitudes toward FP and pregnancy based on various attributes: (i) stage of the disease, (ii) patient gender and (iii) concerns about the welfare of the resulting offspring in AYA populations. Specifically, the following distinctions were present in the scenarios: Stage; localized (A and B) versus metastatic (C and D); gender: males (B and C) versus females (A and D); Presence of concern about the welfare of the resulting offspring (C and D) versus not (A and B) (see Table 1).

**TABLE 1:** Key distinctions between various scenarios.

Scenario	Attributes
Scenario A	Age mid‐30s
	Female gender
	Localized disease
	30%–40% risk of recurrence
	No concern about the welfare of the resulting offspring
Scenario B	Age mid‐20s
	Male gender
	Localized disease
	High cure rate
	No concern about the welfare of the resulting offspring
Scenario C	Age mid‐30s
	Male gender
	Metastatic first line
	Concern about the welfare of the resulting offspring
Scenario D	Age mid‐20s
	Female gender
	Metastatic disease
	Concern about the welfare of the resulting offspring

### 2.3 Survey implementation

Physicians from across Ontario were invited to participate in an electronic survey through a collection of publicly available email addresses. In addition, the survey was sent to all Division Heads of Cancer Centres in Ontario (with a medical oncology training program, six programs total) with a request for the email to be distributed to the oncologists in their cancer centre. A list of oncologists was obtained from each Division Head's administrative team to ensure accurate numbers. A reminder notice was sent out 2 and 4 weeks after the initial invitation. The study was closed for participation 2 months after the initial invitation. The survey was hosted on a secure database, and access to the data was password‐protected. Upon survey closing, data was transferred to and stored on The Ottawa Hospital server that was password‐protected and secured by an institutional firewall. The study was approved by The Ottawa Health Science Network Research Ethics Board. A waiver of informed consent was accepted, as this was an anonymous survey among healthy subjects, with minimal risk of harm to the participants. By answering the survey questions, implied consent was given. Respondents were not compensated.

### 2.4 Data analysis

Categorical data were expressed as frequencies and compared by the Chi‐square test. Binary univariate logistic regression was constructed for the prediction of the effect of baseline demographics. Multivariate analysis was not conducted due to the small sample size. All tests were 2‐sided, and 95% confidence intervals were constructed to determine statistical significance. The analysis was performed using SPSS for Mac (v27; IBM Corp).

## 3 RESULTS

### 3.1 Survey participants

From February 28 to April 30, 2020, survey invitations were sent to 135 medical oncologists and medical oncology fellows and residents. Of the lists provided by Division Heads, no new names were identified; however, some oncologists were identified as retired or on leave. Out‐of‐office responses were received for 44 individuals, resulting in 91 individuals total with confirmed receipt of the survey invitation. Forty individuals completed the survey (response rate 44%). Respondents' baseline characteristics are available in Table 2. Physicians' responses to the factors that might associate with offering fertility preservation referrals prior to cancer therapy are summarized in Table 3.

**TABLE 2:** Participants characteristics.

Characteristics	Number (%)
Gender	
Male	15 (37.5)
Female	25 (62.5)
Marital status	
Single	4 (10)
Married	31 (77.5)
Divorced	2 (5)
Other^b^	2 (5)
NA	1 (2.5)
Parental Status	
Parent	30 (75)
Non‐parent	9 (22.5)
NA	1
Religion	
Non‐religious	12 (30)
Catholic	6 (15)
Jewish	6 (15)
Protestant	5 (12)
Other^c^	7 (17.5)
Prefer not to answer	4 (10)
Years in medical oncology practice	
1–5	13 (32.5)
5–10	7 (17.5)
>10	13 (32.5)
Residents/Fellows	7 (17.2)
Clinical practice setting	
Academic center (oncologist has university appointment)	38 (95)
Community	1 (2.5)
NA	1 (2.5)
Tumor site^a^	
Gastrointestinal	22 (45)
Breast	20 (50)
Lung	12 (30)
Genitourinary	12 (30)
Sarcoma	9 (22.5)
Melanoma	8 (20)
Head and neck	5 (12.5)
Central nervous system	4 (10)
Gynecology	2 (5)
Other	2 (5)
Number of patients referred to fertility preservation in the last 12 months	
1–5	18 (45)
6–10	6 (15)
11–20	9 (22.5)
>20	3 (7.5)

Abbreviation: NA, not available.

^a^ Numbers add up to more than 40, as respondents may treat more than one disease site.

^b^ 1 Common‐law, 1 in a relationship.

^c^ 3 Muslim, 1 Hindu, 1 Sikh, 1 Other, 1 N/A.

**TABLE 3:** Potential factors impacting fertility preservation referrals prior to cancer therapy (physicians' responses).

Is fertility preservation covered or partially covered in the province you work in? (n = 39), n (%)	
Yes	16 (40)
No	11 (26.5)
Unsure	12 (30)
Does the gender of the patient impact whether you offer fertility preservation? (*n* = 36), *n* (%)	
Yes	10 (27.8)
No	26 (72.2)
Do you routinely raise the option of fertility preservation with all your childbearing age patients prior to starting treatment which can impair fertility? (*n* = 36), *n* (%)	
Yes	21 (58.3)
Only for patients prior to receiving potential curative systemic therapy.	13 (36.1)
No	2 (5.6)
Should concerns about the welfare of the resulting offspring be a cause for denying cancer patients assistance in reproduction? (*n* = 35), *n* (%)	
Yes	5 (14.3)
No	14 (40)
It should be discouraged but assistance in reproduction should not be denied.	16 (45.7)
Do you feel comfortable discussing fertility preservation with your patients? (*n* = 35), *n* (%)	
Yes	31 (86.1)
No	5 (13.9)
Do you feel you are up to date on current fertility preservation methods? (*n* = 36), *n* (%)	
Yes	11 (30.6)
No	14 (38.9)
Unsure	11 (30.6)

### 3.2 Provincial insurance coverage for FP

Among respondents, 26.5% thought there was no insurance coverage for FP in the province, 30% were unsure of insurance coverage for FP, and only 40% were aware of insurance coverage for FP. Of the 16 respondents aware of FP coverage, two physicians thought FP coverage was without limitation, three that coverage was limited to co‐pay, one that it was limited to a cap, four that it was limited only to certain scenarios, and six were unsure. There was no association with years of practice (*p* = 0.87) or number of referrals (*p* = 0.12).

### 3.3 Impact of gender

A total of 27.8% of respondents identified that patient gender impacts offering of FP, and 65% identified female patients were referred less often. The reason endorsed was that FP in females is more time‐consuming, and most patients cannot afford to delay their treatment (71%).

### 3.4 Attitudes toward offering FP to AYA patients

A total of 58% of respondents would offer FP to all patients of reproductive age, and 36% only to patients prior to receiving curative intent systemic therapy. There was no association between attitudes for offering FP and physician characteristics assessed, such as years of practice (*p* = 0.83), gender of the physician (*p* = 0.56), physician's number of children (*p* = 0.14), marital status of the physician (*p* = 0.29), religious affiliation of the physician (*p* = 0.43), or tumor site (*p* = 0.49).

### 3.5 Concerns about the welfare of the resulting offspring

A total of 40% of respondents endorsed that concerns about the welfare of the resulting offspring should not be a cause for denying cancer patients assistance in reproduction, 14% endorsed it should be a cause for denial, and 46% endorsed discouraging FP, but assistance in FP should not be denied. There was a statistically significant difference between religiously affiliated versus non‐religiously affiliated physicians (55% vs. 9.1%, *p* = 0.01), with religiously affiliated physicians endorsing that concerns about the welfare of the resulting offspring should not be a cause for denying cancer patients assistance in reproduction. No other associations were noted (tumor site, *p* = 0.48; years of practice, *p* = 0.12; physician gender *p* = 0.67; physician marital status, *p* = 0.55, physician number of children, *p* = 0.66).

### 3.6 Comfortability of discussing FP with patients

Most respondents (86%) felt comfortable discussing FP with the patients. There was no difference based on religious versus non‐religious affiliation (*p* = 0.67), practice sitting (*p* = 0.85), years in practice (*p* = 0.78), marital status (*p* = 0.54), physician number of children (*p* = 0.27), or tumor site (breast cancer, *p* = 0.63; lung cancer, *p* = 0.78; gastrointestinal, *p* = 0.53; head and neck, 0.06). Logistic regression analysis demonstrated that female doctors are more likely to report being comfortable discussing FP (OR: 10.8, 95% CI: 1.2–97.79, *p* = 0.03).

### 3.7 Confidence about being up to date in FP methods

A total of 30.6% of respondents felt up to date on current FP methods, 38.9% felt they were not, and 30.6% were unsure. Logistic regression identified that female respondents (OR 10.8, 95% CI 1.2–97.7, *p* = 0.03) were more likely to feel up to date on FP methods. Physicians treating genitourinary malignancies were less likely to feel up to date (OR 0.04, 95% CI 0.004–0.52, *p* = 0.01). There were no other significant associations identified for confidence in FP knowledge.

### 3.8 Behavior in theoretical scenarios

Scenario A – Part 1: Patient X is a 34‐year‐old female diagnosed with localized (stage III) triple‐negative invasive ductal carcinoma of the breast. She underwent right breast lumpectomy with sentinel node biopsy and recently completed adjuvant chemotherapy (Doxorubicin+Cyclophosphamide and Paclitaxel) and adjuvant radiotherapy. Her estimated 5‐year risk for metastatic disease recurrence is about 30%–40%.

Forty respondents answered this scenario. Most respondents (95%) would refer this patient for FP prior to chemotherapy, and 70% of respondents consistently offer FP in this situation. Thirty‐five (87.5%) would discuss the risks of metastasis with the patient. Twenty‐four (60%) felt FP should be covered by the province in this scenario. Ten (25%) endorsed the risk of developing metastatic disease should not impact offering FP, 23 (57.5%) think it should impact FP, and 6 (15%) were unsure.

Scenario A – Part 2: The patient completed adjuvant treatment and is on routine follow‐up; 6 months after completing treatment, she told you that she is considering pregnancy.

Thirty‐nine respondents answered this scenario. Twenty‐three (59%) would discourage the patient from pregnancy, and 16 (41%) would not discourage pregnancy in this woman. The reasons for discouraging pregnancy were: (i) the risk of developing the metastatic disease in the near future is high, and pregnancy at this point could delay treatment if needed (60%, 14/23), and (ii) concern for the welfare of her future offspring, as her risk for developing metastatic disease and short life expectancy is high (26%, 6/23). The reasons for not discouraging the patient from pregnancy included: (i) it is the patient's rights to decide after discussion (53%, 9/17), and (ii) it is unethical to discourage the patient from considering pregnancy (47%, 8/17). Twenty‐four (61.5%) thought FP should be covered by the province in this situation.

Scenario B – Patient Y is a 26‐year‐old male diagnosed with stage 2 testicular cancer. He is scheduled to start chemotherapy (Bleomycin+Etoposide+Cisplatin) with a curative intent with an expected cure rate above 90%.

Thirty‐nine respondents answered this scenario. All the respondents would offer him a referral for FP; however, 91% (33/36) always offer a referral for FP in such a scenario. Twenty‐nine out of 38 (76.3%) respondents thought the province should cover FP in this scenario.

Scenario C – Patient Y is a 39‐year‐old male diagnosed with metastatic gastric cancer and is about to start first‐line palliative chemotherapy.

Thirty‐eight respondents answered this scenario. Twenty‐three (60.5%) respondents would not offer a referral for FP for this patient, with the predominant reasons being that his prognosis is poor (47.8%, 11/23), it is unfair/unethical to the future offspring (60%, 14/23), and a referral for FP could delay chemotherapy initiation significantly (13%, 3/23). Out of the 15 who would refer this patient for FP, only 9 (60%) do so in practice. In this scenario, 32 (84%) respondents thought the presence of metastatic disease should impact fertility decisions. If this patient were to consider pregnancy, 19 (51%) would not discourage him. Out of the 18 who would discourage him, 72% indicated concern for the welfare of his offspring as his risk for short life expectancy is high. Only 12 out of 36 (33%) felt FP should be covered provincially in this scenario, 13 (36%) felt it should not be, and 11 (31%) were unsure.

Scenario D – Patient Z is a 25‐year‐old female in a long‐term relationship diagnosed with metastatic Ewing sarcoma. She is about to commence second‐line chemotherapy. She and her partner are considering pregnancy using a gestational carrier.

Thirty‐six respondents answered this scenario. Most respondents (72.2%) endorsed the presence of metastatic disease impacts assistance in reproduction decisions, and the rest do not think so. Eighteen respondents (50%) would not discourage the patient, and 50% would discourage the patient from considering pregnancy. Of those that discouraged pregnancy, the majority (77%, 14/18) endorsed concern for the welfare of her offspring due to her short life expectancy as the reason. Eighteen respondents (50%) thought the province should not cover reproduction assistance for this patient, 19% thought the province should cover reproduction assistance, and 31% were unsure.

Table 4 provides a summary of physicians' attitude toward offering FP, supporting patient pregnancy desires and supporting provincial financial assistance in the theoretical scenarios. In theoretical scenarios, respondents were more likely to refer to FP for localized disease, e.g., scenario A (95%), than metastatic disease, e.g., Scenario C (39.5%), *p* < 0.001. Regarding discouraging pregnancy, there was no statistical significance between the localized scenario A (41%) and metastatic scenarios C (49%, *p* = 0.5) or D (50%, *p* = 0.43).

**TABLE 4:** Respondent attitudes toward offering fertility preservation (FP), supporting patient pregnancy desires and supporting provincial financial assistance in theoretical scenarios.

*Scenarios*	Respondents, *n* (%)	Offer FP referral (yes), *n* (%)	Patient considering pregnancy – physician supportive, *n* (%)	Cost of FP covered by province (yes), *n* (%)
*Scenario A – Part 1* (Female, Stage III breast cancer with a high risk of recurrence)	40 (100)	38 (95)	NA	24 (60)
*Scenario A – Part 2, 6 months post‐treatment completion*	39 (97.5)	NA	23 (59)	24 (61.5)
*Scenario B* (Stage II testicular cancer with a high estimated cure rate)	39 (97.5)	39 (100)	NA	29 (74.3)
*Scenario C* (Male, Stage IV Gastric Cancer)	38 (95)	15 (39.5)	19 (51)	12 (33)
*Scenario D* (Female, Stage IV Ewing Sarcoma, second line)	36 (90)	NA	18 (50)	7 (19)

Abbreviation: NA, not applicable.

## 4 DISCUSSION

In the last several decades, a substantial improvement in cancer management has been achieved, which has led to improvements in survival.^12^, ^27^, ^28^ With these advances, the long‐term adverse effects of treatment become an increasing concern. Therefore, counseling patients before treatment regarding fertility impact and providing them with FP options is recommended by scientific societies, including the American Society for Reproductive Medicine,^25^ the American Society of Clinical Oncology (ASCO),^13^, ^14^, ^15^ the National Comprehensive Cancer Network (NCCN),^29^ and the European Society for Medical Oncology (ESMO).^30^

Herein, we provide up‐to‐date medical oncologists' attitudes toward FP and pregnancy in cancer patients and, for the first time, include high‐risk cancers for recurrence and metastatic disease. We found that 58% of respondents offer FP for all patients, and one‐third offer FP only for patients receiving curative‐intent chemotherapy. 40% of respondents endorsed that concern about the welfare of the resulting offspring should not be a cause for denying cancer patients assistance in reproduction. However, in the theoretical scenarios, physicians were more likely to refer to FP for localized disease (92.8%–100% of respondents) than metastatic disease (39.5%–50% of respondents).

Ninety percent of respondents had experience referring patients to FP in this study, which is an improvement from previously reported Canadian data, of 66% and 80% for female and male patients, respectively.^31^ Approximately one‐fourth (27.8%) of respondents reported that patient gender impacts their decision to offer FP, and the majority stated female patients were referred less often because FP methods in females are more time‐consuming. This is consistent with previously reported literature. A Swedish study reported that the male and female patients who received FP assistance were 48% versus 14%, respectively.^22^ Another study from California also reported that only 12.2% of women received counseling regarding FP.^23^ Conversely, a recent survey for nearly seven thousand patients of reproductive age showed that females were more likely than men to be counseled regarding FP before chemotherapy (56% vs. 32%).^32^ In those who received counseling, the information was sufficient, yet improvement in communication and assistance in shared decision‐making was recommended.^33^

Once disease stage was raised, differences in responder attitudes emerged in this work. The predominant reason reported was the welfare of the future offspring, with some physicians reporting active discouragement of procreation. International bodies have clearly endorsed that regardless of type and stage of cancer, patients of reproductive age should participate in FP discussions,^34^ and welfare for offspring should not be a reason for declining assistance for FP.^25^ Discussions regarding FP are distinct from referrals for FP; nonetheless, the occurrence of FP discussions with appropriate documentation is paramount. However, the complex decision‐making individuals with advanced cancer face is compounded in the AYA demographic. The literature consistently demonstrates that AYA requires clinicians to^1^: have clear communication about prognosis,^2^ take the time to connect with patients and elicit their values, and^3^ share decision‐making with evidence to support clinical decisions and acceptance of patient preferences and choices.^35^ The emotional difficulties health care providers (HCPs) may have when providing care to AYA with advanced cancer may affect the discussion and options presented.^36^ Further supports for patients and HCPs are necessary, as corroborated by international recommendations.^15^, ^29^, ^30^, ^37^, ^38^ HCPs, for example, could benefit from targeted education strategies that include FP guidelines update, in addition to recent ethical guideline updates and techniques on how to elicit and integrate patient preferences. In addition, although oncologists may not be confident in current FP methods, it is more important that they know how and where to refer patients to appropriate specialists for accurate information, as opposed to maintaining this knowledge base.

The urgency of treatment was reported as one barrier to FP in this study. However, the time to assessments in Canada should be less concerning than noted in this work. A national survey in 2012 demonstrated that fertility centers in Canada prioritize timely oncology patient assessments; out of 25 centers, 64% assess patients within 3 days, 16% within 1 week, and the rest within 3 weeks.^39^ However, the subsequent time to FP treatment is unknown. Various cancer centres many need increased education of available resources and local supports. Alternate reasons for lack of FP referral could also include a patient not wishing to have biological children in the future, the limited gonadotoxicity potential of the proposed cancer therapy, physical inability to attend an FP appointment or potential lack of available FP options. For example, ovarian tissue freezing in Canada is not currently available outside of clinical trials.

Improving knowledge is corroborated by only 40% of respondents being aware of public insurance coverage for FP. Of these respondents, all were accurate in their knowledge of provincial coverage. FP is funded in Ontario through the Ontario Fertility Program for patients who receive chemotherapy and are aged less than 43 years once per lifetime. The FP of additional batches of ovum or sperm is not covered.^40^ Additionally, financial support is available by Fertile Future.^41^ Notably, 61%–76% of physicians felt the FP should be publicly covered for curative intent therapy; only 33% felt this coverage should apply to patients undergoing palliative intent treatments. This raises concerns about equity, and also highlights the balance clinicians aim to strike between advocating for patients, weighing overall risks and benefits to patients, yet acting as stewards of finite resources in publicly funded systems. The federal *Assisted Human Reproduction Act* introduced in 2004 mandates reproductive tissue be used with the donor's free and informed written consent, thus enabling post‐mortem access to reproductive tissue.^42^ In the context of an AYA patient's health care provider (HCP), we advocate that shared decision‐making that supports the patient's wishes while advising of the objective risks and benefits be undertaken.

This work has limitations. Due to the undertaking of this study at the start of the COVID‐19 pandemic, HCP responses were less than anticipated due to high out‐of‐office rates. This lower response rate is likely due to increased HCP clinical workload and increased personal stresses that all HCPs faced during the pandemic. The decision was made to close the study to further response, as the length of the pandemic was unknown. However, this data highlights the difference in physicians' attitudes toward FP for patients facing curative intent versus palliative intent treatment. Additional survey responses were unlikely to change this outcome. The theoretical scenarios included in this manuscript include all the information respondents received. Given the limited information, room for interpretation and variance may impact results. Attempts to maximize face and content validity were made with pilot surveys' testing and feedback integration to improve survey clarity. However, selection bias may have impacted study results, as most responders (95%) were working in academic settings, and females were more like to respond than males. In addition, respondents may have been more interested or knowledgeable in the subject material. The development of validated tools to measure attitudes toward FP may be one way to address potential biases.

Further research to explore these findings, including qualitative studies, can increase understanding of HCP thought processes and challenges faced. Qualitative studies of patients should also be undertaken to ensure patient perspectives are integrated into future educational efforts.

## 5 CONCLUSION

Among Canadian physicians, there is increased awareness of FP than in previous reports. However, there is a significant difference in physician attitude toward offering FP based on the cancer stage. Increased awareness of standard of care guidelines and resources for difficult situations may improve the frequency of FP discussions for AYAs with a high risk for recurrence or advanced cancer.

## AUTHOR CONTRIBUTIONS

AS and IK generated the idea, AS and IK wrote the proposal, and AS, SA, and IK collected the data. All authors analyzed the data. BA wrote the first draft. All authors contributed to revisions, read the manuscript, and approved the submitted version.

## FUNDING INFORMATION

There are no funding sources to report.

## CONFLICT OF INTEREST

The authors report no conflicts of interest for this work.

## ETHICS STATEMENT

The study was approved by The Ottawa Health Science Network Research Ethics Board. A waiver of informed consent was accepted, as this was an anonymous survey among healthy subjects, with minimal risk of harm to the participants.

## Supporting information


Appendix S1

## Data Availability

Not applicable.
