# Supplementary material for: Perceptions and attitudes of medical oncologists regarding fertility preservation and pregnancy in high‐risk cancer patients: A survey among Canadian medical oncologists
Source: Cancer Med. 2022 Jul 14;12(2):1912–21. doi: 10.1002/cam4.5023 (PMC9883564; doi:10.1002/cam4.5023)
Supplement: Supplementary file 1 — Appendix S1 [file CAM4-12-1912-s001.docx]

**Supplementary 1. The Survey Questions**

Assessment of survey eligibility:

1. Are you a medical oncologist, medical oncology resident or a medical oncology fellow?

A. Yes

B. No

If you answered no to this question you can finish the survey here.

1. Years practicing as a medical oncologist (post residency)
2. 1-5
3. 6-10
4. >10.
5. I am a medical oncology resident
6. I am a medical oncology fellow
7. What is your gender?

A. Male.

B. Female.

C. Transgender.

D. Other.

E. Prefer not to answer.

1. What is your marital status?
   1. Single
   2. Married
   3. Common-law
   4. In a relationship
   5. Widowed
   6. Divorced
   7. Prefer not to answer
2. Do you have children?
   1. Yes
   2. No
   3. Prefer not to answer
3. What is your religious background?
   1. Non-religious
   2. Catholic.
   3. Protestant.
   4. Muslim.
   5. Hindu.
   6. Sikh.
   7. Buddhist.
   8. Jewish (observant).
   9. Jewish (non-observant).
   10. Other.
   11. Prefer not to answer.
4. What is the setting of your clinical practice?

A. Academic (university affiliated)

B. Community practice

1. What tumor sites are you treating in your practice? (select all that apply)
2. Breast
3. Lung
4. GI
5. GU
6. CNS
7. Sarcoma
8. Melanoma
9. Head and neck
10. Other
11. How many patients you have referred for fertility preservation in the last year?
    1. None.
    2. 1-5.
    3. 6-10.
    4. 11-20.
    5. More than 20.
12. In the last year, please estimate how many of your new consults patients were less than 40 years old.

A. None

B. 1-5

C. 6-10

D. 11-20

E. More than 20

1. Is fertility preservation covered or partially covered in the province you work in?
   1. Yes
   2. No
   3. Unsure
2. If you answered Yes (A) in Q. 11 - Are there limitations to the coverage?
   1. No
   2. Yes, there is a co pay
   3. Yes, there is a cap
   4. Yes, only certain scenarios
   5. Unsure.

For the following questions please consider the following scenario (scenario A): Patient X is a 34-year-old female diagnosed with localized (stage 3) triple negative invasive ductal carcinoma of the breast. She underwent right breast lumpectomy and recently completed adjuvant chemotherapy (Doxorubicin+Cyclophosphamide and Paclitaxel) and adjuvant radiotherapy. Her estimated 5 year risk for metastatic disease recurrence is about 30-40%.

1. Would you discuss and offer a referral for fertility preservation prior to starting chemotherapy?
   1. Yes
   2. No
2. If you answered Yes (A) in Q. 13 – Do you always discuss and offer a referral for fertility preservation prior to starting chemotherapy in such a scenario (scenario A)?
   1. Yes.
   2. Only if the planned treatment has a high risk for causing infertility.
   3. Only if it won’t delay significantly adjuvant treatment initiation.

D. Only if the patient raises the issue.

E. Other.

1. If you answered No (B) in Q. 13 – What was your reason for choosing No (B) in Q. 13 (choose all that apply)?

A. The planned treatment should not impair her fertility and therefore there is no need for a referral.

B. I’m not familiar enough with current fertility preservation techniques and therefore I do not feel comfortable enough to discuss this subject.

C. Due to my workload / time constraints.

D. A referral for fertility preservation could delay significantly adjuvant treatment initiation.

E. Current fertility preservation techniques may increase her cancer proliferation/disease progression.

F. Her prognosis is poor and it’s unfair/unethical to the future offspring.

G. Due to financial costs for the patient associated with fertility preservation.

H. I assume that the patient is not interested in fertility preservation referral.

I. Other.

The patient completed adjuvant treatment and is on routine follow up, 6 months after completing treatment, she told you that she is considering pregnancy.

1. Should the risk of developing metastatic disease (with a shorter life span) in the future impact fertility decisions?

A. Yes.

B. No.

C. Unsure.

1. Would you discuss at this point the risks of developing metastatic disease?
   1. Yes
   2. Only if the patient will ask.
   3. No.

1. Would you caution the patient against considering pregnancy at this time?
   1. No.
   2. Yes.
2. If you answered No (A) in Q.18 – What was your reason for choosing No (A) in the previous question (Q.18) (choose all that apply)?

A. There is no reason to caution the patient against pregnancy in this scenario.

B. I do not feel comfortable discussing this subject with the patient.

C. The physician has no role in advising the patient in this subject.

D. It’s unethical to discourage the patient from considering pregnancy.

E. Other.

1. If you answered Yes (B) in Q.18 – What was reason for choosing Yes (B) in the previous question (Q.18) (choose all that apply)?

A. Pregnancy at this point may increase her risk of developing metastatic disease.

B. Her risk of developing metastatic disease in the near future is high and pregnancy at this point could delay treatment if needed.

C. The treatments she received (Doxorubicin+Cyclophosphamide and Paclitaxel chemotherapy) could harm the future embryo / offspring.

D. Concern for the welfare of her future offspring, as her risk for developing metastatic disease and short life expectancy is high.

E. Other.

21. Should fertility preservation be covered by the province in this scenario (scenario A):

A. Yes

B. No

C. Unsure

For the following questions please consider the following scenario (scenario B): Patient Y is a 26 year old male diagnosed with stage 2 testicular cancer. He is scheduled to start chemotherapy (Bleomycin+Etoposide+Cisplatin) with a curative intent with an expected cure rate of above 90%.

22. Would you discuss and offer a referral for fertility preservation prior to starting chemotherapy?

- 1. Yes
  2. No

23. If you answered Yes (A) in Q. 22 – Do you always discuss and offer a referral for fertility preservation prior to starting chemotherapy in such a scenario (scenario B)?

1. Yes.
2. Only if it won’t delay significantly treatment initiation.
3. Only if the patient raises the issue.
4. Other.

24. If you answered No (B) in Q. 22 – What was your reason for choosing No (B) in Q. 22 (choose all that apply)?

A. The planned treatment should not impair his fertility and therefore there is no need for a referral.

B. I’m not familiar enough with current fertility preservation techniques and therefore I do not feel comfortable enough to discuss this subject.

C. Due to my workload / time constraints.

D. A referral for fertility preservation could delay significantly treatment initiation.

E. Current fertility preservation techniques may increase his cancer proliferation/disease progression.

F. His prognosis is poor and it’s unfair/unethical to the future offspring .

G. Due to financial costs for the patient associated with fertility preservation.

H.I assume that the patient is not interested in fertility preservation referral.

I. Other.

25. Should fertility preservation be covered by the province in this scenario (scenario B):

A. Yes

B. No

C. Unsure

For the following questions consider the following scenario (scenario C): Patient Y is a 39 year old male diagnosed with metastatic gastric cancer and is about to start first line palliative chemotherapy.

26. Would you discusses and offer a referral for fertility preservation prior to chemotherapy?

A. Yes.

B. No.

27. If you answered Yes (A) in Q. 26 – Do you always discuss and offer a referral for fertility preservation prior to starting chemotherapy in such a scenario (scenario C)?

A. Yes.

B. Only if the planned treatment has a high risk for causing infertility.

C. Only if it won’t delay significantly chemotherapy initiation.

D. Only if the patient raises the issue.

E. Other.

28. If you answered No (B) in Q. 26 – What was your reason for choosing No (B) in Q. 26 (choose all that apply)?

1. The planned treatment should not impair his fertility and therefore there is no need for a referral.
2. I’m not familiar enough with current fertility preservation techniques and therefore I do not feel comfortable enough to discuss this subject.
3. Due to my workload / time constraints.
4. A referral for fertility preservation could delay significantly chemotherapy initiation.
5. Current fertility preservation techniques may increase his cancer proliferation/disease progression.
6. His prognosis is poor and it’s unfair/unethical to the future offspring.
7. Due to financial costs for the patient associated with fertility preservation.
8. I assume that the patient is not interested in fertility preservation referral.
9. Other.

The patient underwent sperm preservation prior to starting chemotherapy. 2 months after starting chemotherapy the patient and his partner advise you that they are considering pregnancy.

29. Should the presence of metastatic disease (with a shorter life span) impact fertility decisions?

A. Yes.

B. No.

30. Would you caution the patient against considering pregnancy at this time?

A. No.

B. Yes.

31. If you answered No (A) in Q.30 – What was your reason for choosing No (A) in the previous question (Q.30) (choose all that apply)?

A. There is no reason to caution the patient against pregnancy in this scenario.

B. I do not feel comfortable discussing this subject with the patient.

C. The physician has no role in advising the patient in this subject.

D. Other.

32. If you answered Yes (B) in Q.30 – What was your reason for choosing Yes (B) in the previous question (Q.30) (choose all that apply)?

A. Concern for the welfare of his offspring as his risk for short life expectancy is high.

B. Gastric cancer at a young age may raise the suspicious for germline mutation.

C. Other.

33. Should fertility preservation be covered by the province in this scenario (C):

A. Yes

B. No

C. Unsure

For the following questions consider the following scenario (scenario D): Patient Z is a 25-year-old female in a long-term relationship diagnosed with metastatic Ewing sarcoma. She is about to commence second line chemotherapy. She and her partner are considering pregnancy using a gestational carrier.

34. Should the presence of metastatic disease (with a shorter life span) impact assistance in reproduction decisions?

A. Yes.

B. No.

35. Would you caution the patient against considering pregnancy at this time?

A. No.

B. Yes.

36. If you answered No (A) in Q.35 – What was your reason for choosing No (A) in the previous question (Q.35) (choose all that apply)?

A. There is no reason to caution the patient against pregnancy in this scenario.

B. I do not feel comfortable discussing this subject with the patient.

C. The physician has no role in advising the patient in this subject.

D. Other.

37. If you answered Yes (B) in Q.35 – What was your reason for choosing Yes (B) in the previous question (Q.35) (choose all that apply)?

A. Concern for the welfare of his offspring as his risk for short life expectancy is high.

B. Other.

38. Should assistance in reproduction be covered by the province in this scenario (D):

A. Yes

B. No

C. Unsure

39. Do you routinely raise the option of fertility preservation with all your childbearing age patients prior to starting treatment which can impair fertility?

A. Yes.

B. Only for patients prior to receiving potential curative systemic therapy.

C. No.

40. Does the gender of the patient impact whether you offer fertility preservation?

A. Yes

B. No

41. If you answered yes (A) in the previous question (Q.37)- Which patient population is referred less often

A. Females are referred less often

B. Males are referred less often

42. If you answered “Females” (A) in Q.38 - Why do you refer less often females? (choose all that apply).

A. Procedures are not covered

B. Procedures are more complicated

C. The evidence for fertility preservation in females is less robust

D. Fertility preservation in females is less successful.

E. I am not familiar enough with current fertility preservation techniques in females.

F. Fertility preservation in females is more time consuming and most patients can’t afford delaying their treatment.

G. Other.

43. If you answered “Males” (B) in Q.38 - Why do you refer less often males? (choose all that apply)

A. Procedures are not covered

B. Procedures are more complicated

C. The evidence for fertility preservation in males is less robust

D. Fertility preservation in males is less successful.

E. I am not familiar enough with current fertility preservation techniques in males.

F. Fertility preservation in males is more time consuming and most patients can’t afford delaying their treatment.

G. Other.

44. Should concerns about the welfare of the resulting offspring be a cause for denying cancer patients assistance in reproduction?

A. Yes.

B. No.

C. It should be discouraged but assistance in reproduction should not be denied.

45. Do you feel comfortable discussing fertility preservation with your patients?

A. Yes.

B. No.

46. Do you feel you are up to date on current fertility preservation methods?

1. Yes.
2. No.
3. Unsure.

End of the Survey
